# Caring for Families of Patients With Acquired Brain Injury Early During Hospitalization: A Feasibility Study of the SAFIR^©^ Intervention

**DOI:** 10.1177/10748407241270034

**Published:** 2024-09-13

**Authors:** Véronique de Goumoëns, Krystel Bruyere, Dionys Rutz, Jérôme Pasquier, Yann-Olivier Bettex, Krystel Vasserot, Philippe Ryvlin, Anne-Sylvie Ramelet

**Affiliations:** 1 La Source School of Nursing, HES-SO, University of Applied Sciences and Arts, Western Switzerland, Lausanne, Switzerland; 2 Lausanne University Hospital, (CHUV), Switzerland; 3 Faculty of Biology and Medicine, Institute of Higher Education and Research in Healthcare- IUFRS, University of Lausanne, Lausanne, Switzerland; 4 Center for Primary Care and Public Health (Unisanté), University of Lausanne, Lausanne, Switzerland

**Keywords:** family nursing intervention, feasibility study, acute care, early intervention, acquired brain injury

## Abstract

This study aimed to assess the feasibility of a complex family nursing intervention (SAFIR©) designed to support families of patients with acquired brain injuries during the early phase of hospitalization, using a one-group pre- and post-test design with a one-month follow-up. Family members participated in four family meetings. Quantitative data were collected using an intervention protocol checklist and questionnaires. Qualitative data were gathered through semi-structured interviews, written open-ended questions, and note-taking. Feasibility outcomes revealed a family recruitment rate of 15.4% and a retention rate of 100%. Protocol adherence ranged from 94% in Phase 1 to 78% in Phase 3. Our results indicated that the intervention was meaningful and suitable for family members (n=7), healthcare provider (n=1), and nursing managers (n=6). From a sustainability perspective, our findings suggest the need to formally involve the entire inter-professional team in the intervention. Further evaluation of the intervention is warranted through a large-scale experimental.

## Background

Acquired brain injuries (ABIs) are the leading causes of disability and mortality in the adult population globally (Feigin et al., 2019; Vos et al., 2020). ABIs yield far-reaching and enduring repercussions for affected individuals, their families, and the broader community. The intricate interplay of physical, cognitive, and behavioral changes at the individual level profoundly impacts on the entire family unit. Families grapple with heightened emotional intensity, social isolation, and complex family dynamics (Fisher et al., 2020). Families of patients with ABI generally perceive high caregiving burden, often exceeding that observed in the context of neurological pathologies (Tramonti et al., 2019). While most families wish to engage with health care professionals (HCPs), they necessitate additional support tailored to their needs from the onset of injury, throughout the hospital stay to post-hospital discharge in the community setting (de Goumoëns et al., 2019).

Nurses and other health care providers are responsible for supporting families as units of care (International Family Nursing Association [IFNA], 2017). Family nursing interventions are defined as time-limited interventions that target the family as the “unit of intervention” and take the form of a collaborative, non-hierarchical interaction between a family and an interprofessional team in which one or more nurses lead and/or deliver the intervention (Eustace et al., 2015). These types of interventions have been shown to be effective in adults with specific chronic conditions and their families (Chesla, 2010). Positive effects on readmission rates, emergency department visits, and family anxiety have been demonstrated (Deek et al., 2016). The results of a scoping review that synthetized the evidence on interventions for patients with ABI demonstrated a growing interest in considering ABI as a family issue but also showed a high heterogeneity in the interventions provided (de Goumoëns et al., 2018); the findings failed to identify any family intervention with proven efficacy in the acute phase for this population. In the absence of substantiated evidence delineating optimal family nursing interventions for individuals with ABI during the acute hospitalization phase, a new intervention, the SAFIR^©^ intervention, has been developed (de Goumoëns et al., 2022). Considering its multifaceted nature, testing the feasibility of the intervention was deemed necessary before it can be tested in a future definitive trial (Craig et al., 2013). This undertaking is relevant, given the growing recognition of the central role of family interventions within clinical neuroscience settings (Hinkle et al., 2022). The aim of this study was to appraise the feasibility of SAFIR^©^, a newly developed family intervention to support families of ABI patients, from the early phases of hospitalization, as a prelude to a forthcoming large-scale study.

## Method

### Design

This feasibility study employed a one-group pre- and post-test design with a 1-month follow-up. The authors adhered to the CONSORT 2010 statement: extension to randomized pilot and feasibility trials (Eldridge et al., 2016). The study was approved by the human research ethics committee (CER-VD 2019-00825) and has been registered on clinicaltrial.gov (NCT04138524) as well as in the National Clinical Trial Portal (SNCTP000003405).

### Study Settings and Participants

This study was conducted in the clinical neuroscience department of a university hospital, which encompasses three wards specializing in neurology, neurosurgery, and acute neurorehabilitation, collectively providing a total of 50 acute care beds. The study participants included patients, their family members, the intervention provider, and the nursing managers (NMs). Patients were eligible if they met the following criteria: age ≥18 years, a medical diagnosis of moderate-to-severe ABI as defined by the National Institutes of Health Stroke Scale (NIHSS) (Ortiz & Sacco, 2014) or the Glasgow Coma Scale (Teasdale et al., 2014), and hospitalization for a minimum of 1 day and a maximum of 2 days in the department at the time of screening. Patients were excluded if they had a medical diagnosis of mild ABI due to significant differences in physical, cognitive, or behavioral impact, and differences in clinical outcomes such as length of stay and hospital discharge. Family members were eligible if they were: a relative of the patient, a significant other, the legal representative of the patient, aged ≥18 years, and fluent in French. A convenience sample of 10 families has been estimated, as no sample size calculation was required for this feasibility study (Bowen et al., 2009). The intervention provider was eligible if: holding a master’s degree in nursing sciences and trained and expert in Family Systems Nursing and Neurology. NMs were eligible if they have a responsibility in units where the SAFIR^©^ intervention was tested.

### Intervention

SAFIR^©^ (Soins Aux Familles: Intervention pRécoce [Care For Families: Early Intervention]), is a nurse-led complex intervention that was developed using the initial phase of the Medical Research Council Framework ref. Grounded in the Family Systems Nursing (FSN) theory (Schober & Affara, 2001), specifically the Calgary Family Assessment and Intervention Models (Shajan & Snell, 2019), SAFIR^©^ comprises family meetings delivered in three phases (days 2, 5, and 10) with a 1-month follow-up. It aims to provide family assessment, emotional support, personalized information delivery, family involvement in care, and facilitate interprofessional collaboration. The intervention aims to establish and sustain a genuine relationship, facilitate transition, and maintain ongoing contact with the family. A standardized manual was developed for intervention delivery, and it was decided that only a Clinical Nurse Specialist (CNS) with neuroscience expertise should provide the intervention following specific FSN training. The determinants of the effectiveness of the SAFIR intervention were selected according to the FSN theoretical framework: family functioning, coping, and support perceived by families from the nursing team. Detailed information on the intervention and the logic model are available in a prior publication (de Goumoëns et al., 2022).

### Measures

#### Feasibility of the SAFIR^©^ Intervention

In accordance with Bowen criteria (Bowen et al., 2009), feasibility was assessed across multiple dimensions: (a) acceptability (evaluating family satisfaction, retention rates, and perceived appropriateness of the intervention), (b) implementation (measuring fidelity to the protocol and the required resources), (c) practicality (assessing the provider’s ability to execute the intervention), (d) integration (evaluating compatibility with existing care infrastructure, perceived sustainability, and cost estimation based on the duration of the intervention), and (e) expansion (assessing alignment with organizational goals and culture). The following instruments were utilized to evaluate feasibility outcomes: an intervention protocol checklist, written open-ended questions for family members, a notebook for the CNS, and semi-structured interviews with the CNS and NMs.

An intervention protocol checklist derived from the intervention manual was employed to gather data regarding protocol adherence (implementation), cost analysis (integration) by tracking the duration of family meetings, and recruitment and retention rates (acceptability). Family members responded to four open-ended questions to assess acceptability and integration: *How did you experience this intervention? What aspects of this intervention were the most helpful for you and your family? What were the primary challenges you encountered during the intervention? If this intervention was to be repeated, what suggestions for improvement would you suggest?* The CNS used a notebook to record reflections regarding positive and negative aspects of each phase, suggestions for improvement, challenges, and any additional comment. For semi-structured interviews, an interview guide was developed by the research team, with questions aligned with Bowen’s definition of feasibility outcomes. The interview guide and the objectives pursued are listed in Table 1.

**Table 1.** Interview Guide and Objectives.

Theme	Exploration of feasibility outcomes
Experience and satisfaction regarding the SAFIR^©^ intervention (role and responsibilities in the process)	Satisfaction with the intervention (acceptability)
The benefits of the SAFIR^©^ intervention for the population, the department, and the health professionals	Perceived appropriateness (acceptability)
Integration of the SAFIR^©^ intervention in the standard care of the department and suggestion for improvement (structure and modalities)	Ability of the provider to carry out the intervention (practicality) Fit with organizational goals and culture (expansion)
Implementation and transferability of the SAFIR^©^ intervention in other contexts of the department (barriers and benefits)	Perceived sustainability (integration)

#### Family Functioning Assessment

Family functioning was evaluated using the validated Iceland-Expressive Family Functioning Questionnaire (ICE-EFFQ) (Sveinbjarnardottir et al., 2012a), a 17-item instrument encompassing four dimensions: expressive emotions, collaboration and problem solving, communication, and behavior. Responses were rated on a 5-point Likert-type scale, ranging from 1 (*almost never*) to 5 (*all the time*), reflecting each family member’s perception of the illness’s impact. Scores ranged from 17 to 85, with higher scores indicating better family functioning (no threshold was established to distinguish between optimal and suboptimal family functioning). This questionnaire has established validity and reliability with a Cronbach’s α = .912 (Sveinbjarnardottir et al., 2012a). The ICE-EFFQ was translated in French using translation guidelines (Wild et al., 2005).

#### Family Perceived Support Assessment

Perceived support was evaluated using the validated Iceland-Family Perceived Support Questionnaire (ICE-FPSQ) (Sveinbjarnardottir et al., 2012b), a 14-item instrument assessing two dimensions of perceived support: (a) cognitive and (b) emotional. Utilizing a 5-point Likert-type scale, ranging from 1 (*almost never*) to 5 (*all the time*), the questionnaire measured each family member’s perception of cognitive and emotional support provided by nurses. Scores varied between 14 and 70, with higher scores indicating better perceived support (no cutoff score was established to differentiate between adequate and inadequate nurse support). This questionnaire has established validity and reliability with a Cronbach’s α = .959 (Sveinbjarnardottir et al., 2012b). It was translated in French using translation guidelines (Wild et al., 2005).

#### Family Coping Assessment

Family coping was evaluated utilizing the French version of the Brief COPE inventory developed by Carver and subsequently translated, adapted, and validated into French (Carver, 1997; Muller & Spitz, 2003). The 14 subscale Brief COPE inventory comprises 28 items rated on a 4-point Likert-type scale, ranging from 1 (*I haven’t been doing that at all*) to 4 (*I have been doing this a lot*). The 14 subscales encompass: (a) active coping, (b) planning, (c) using instrumental support, (d) using emotional support, (e) venting, (f) behavioral disengagement, (g) self-distraction, (h) self-blame, (i) positive reframing, (j) humor, (k) denial, (l) acceptance, (m) religion, and (n) substance use. The results yield two distinct coping styles: (a) Approach Coping, derived from the six subscales of active coping, positive reframing, planning, acceptance, using emotional support, and using instrumental support and (b) Avoidant Coping, calculated from the six subscales of denial, substance use, venting, behavioral disengagement, self-distraction, and self-blame.

### Procedure

**Figure 1.**
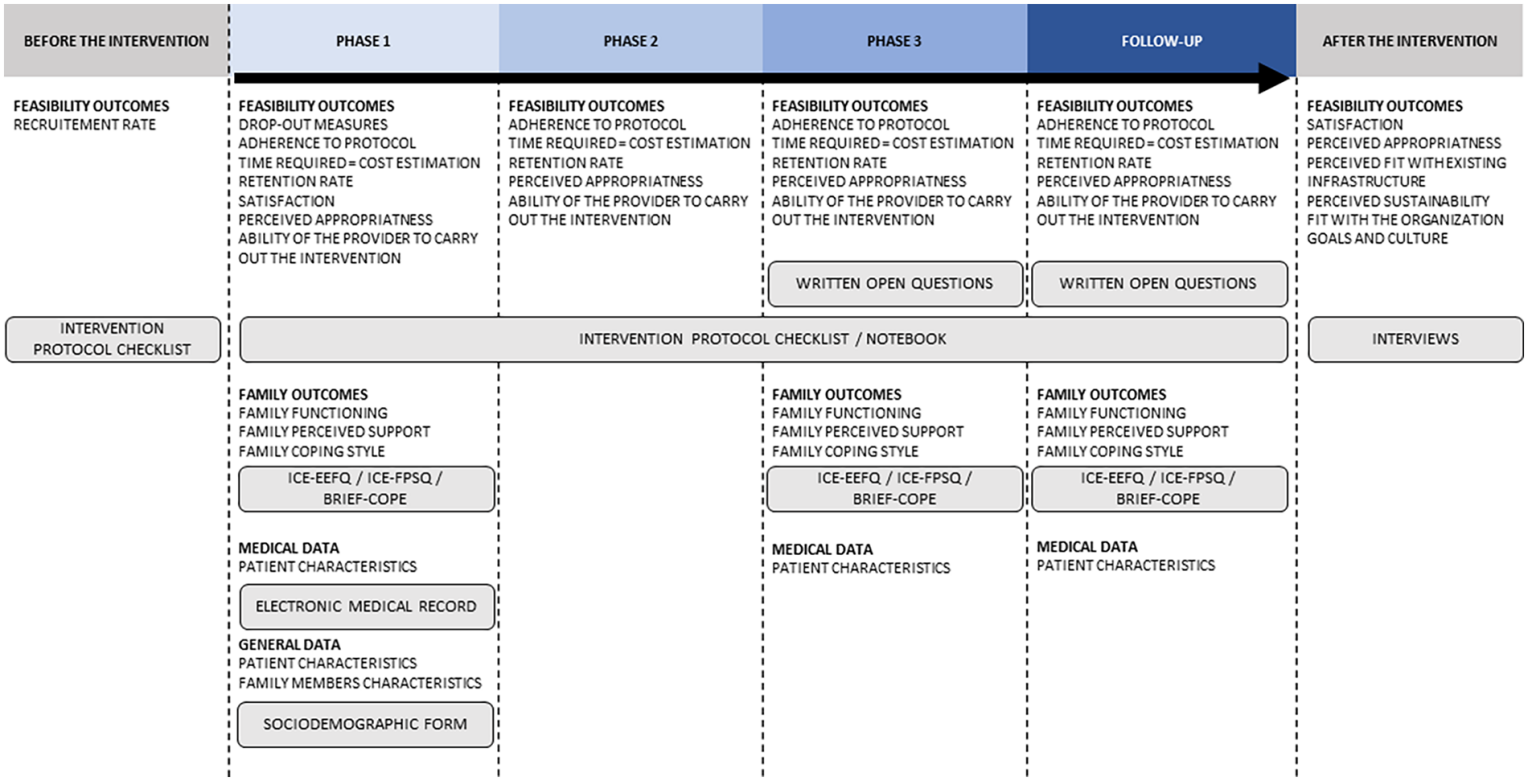
Structure of the Data Collection.

Patient and family member were recruited sequentially, starting with patients’ screening and consent, followed by family members. When patients were unable to provide informed consent, family members were directly approached for consent. Eligibility assessments for patients were conducted daily by the unit’s head nurse, initiated on day 1 (D1) of the patient’s admission to the department. Eligible patient and family members were invited to participate in the study by the intervention provider and obtain their consent. Data collection involved measurements at various stages, with a detailed data collection framework provided in Figure 1.

### Data Analysis

#### Quantitative Analysis

Descriptive statistics, such as means, medians, and standard deviations, were computed using STATA^®^ Release 15 (StataCorp, 2019).

#### Qualitative Analysis

Qualitative data from open-ended family member responses, intervention provider notes, and semi-structured interviews with NMs and CNS were subjected to inductive and deductive content analysis (Mayring, 2014), using MAXQDA 18.0 software (VERBI Software, 2016).

## Data Coding and Analysis

Two researchers independently coded the data, with a third researcher resolving coding disagreements. Initial deductive analysis using categories aligned with Bowen’s key feasibility outcomes (Bowen et al., 2009): acceptability (assessed through satisfaction and perceived appropriateness), practicality (evaluated based on the provider’s ability to implement the intervention), integration (examined for perceived sustainability), and expansion (assessed in relation to alignment with organizational goals and culture). Inductive analysis identified nine subcategories, each corresponding to at least one of the main categories. These subcategories encompassed challenges related to the intervention, family needs, care environment, beliefs, HCPs needs, transferability, proposals for new modalities, vision, and interprofessional collaboration. The coding matrix was jointly revised after analyzing 15% of the data set, with validation of categories and subcategories by the research team. The final coding matrix comprised five main categories and nine subcategories. To ensure the scientific rigor of the qualitative analysis, recommendations from Lincoln and Guba (1985) were followed: data credibility was ensured by frequent meetings between the principal investigator and the research team; data confirmability was ensured by an analysis process carried out by an interdisciplinary team, including a nurse, physical therapist, and occupational therapist, who reviewed the analysis to gain a thorough and complete understanding of the data and minimize the risk of bias; data dependability was ensured by the transparency of the data collection and analysis process; data collection was carried out until there were no new elements in the interviews (data saturation); and data transferability was ensured by a detailed description of the participants’ experiences.

## Results

### General Data

#### Participant Characteristics

Five families, comprising five patients and seven family members, participated in the study. Table 2 presents the characteristics of these patients and family members. All participants suffered from stroke. The level of impairment was thus assessed with the NIHSS (Ortiz & Sacco, 2014) and the clinical judgment of the HCP.

**Table 2.** Characteristics of Patients and Family Members.

Characteristics	Patients	Family members
Age *Mdn (IQR) (n=5)/(n=7)*	79.8 (8.2)	64.4 (14.8)
Length of stay *Mdn (IQR)*	14 (9)	NA
Gender *n (%)*		
Male	3 (60)	3 (40)
Female	2 (40)	4 (60)
Injury type *n (%)*		
Stroke	5 (100)	NA
Severity of ABI *n (%)*		
Moderate	3 (60)	NA
Severe	2 (40)	NA
Level of impairment D1 *n (%)*		
Moderate	3 (60)	NA
Severe	2 (40)	NA
Level of impairment D10 *n (%)*		
Moderate	3 (60)	NA
Severe	2 (40)	NA
Level of impairment D30 *n (%)*		
Moderate	4 (1)	NA
Education level *n (%)*		
High school diploma	1 (20)	1 (20)
College graduate	2 (40)	4 (60)
Postgraduate degree	2 (40)	2 (30)
Professional activity *n (%)*		
Retired	5 (1)	4 (60)
Active	0 (0)	3 (40)
Relationship with the patient *n (%)*		
Spouse/partner		3 (40)
Child		4 (60)
Sharing home *n (%)*		4 (60)

#### Intervention Provider’s and Nurse Managers Characteristics

The intervention provider was a CNS with over 8 years of nursing experience, with 4 years specifically within the department, holds a master’s degree in nursing, and has undergone training specific to the SAFIR^©^ intervention. Five NMs with over 5 years of practice, recognized expertise in the field, and managerial roles in the department participated in the study. Their roles ranged from community manager to departmental director of care. Three of them were actively involved in the training and deployment of SAFIR in the unit, while the other 2 were very active in recruiting families.

### Quantitative Findings

#### Recruitment and Retention Rate

**Figure 2:**
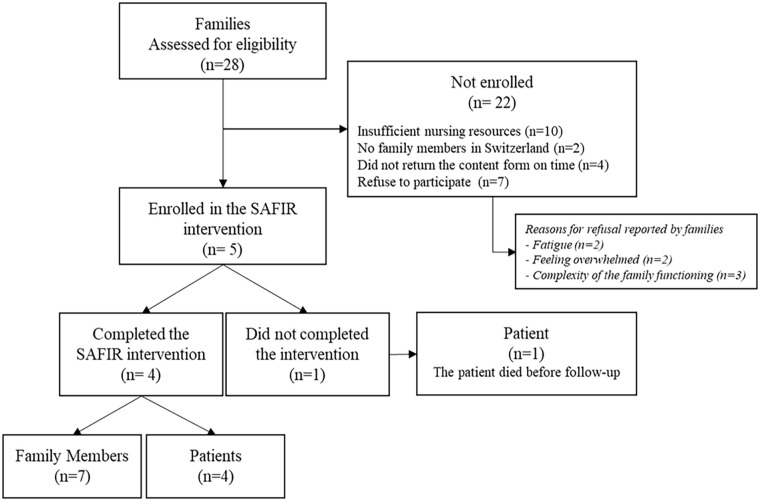
Participants Flowchart.

The recruitment of patients and families took place from November 1, 2019 to August 31, 2020, with a temporary interruption during the initial wave of the SARS-CoV-2 pandemic (March to July 2020). Out of all eligible patients and during the SARS-CoV-2 pandemic with limited family access due to visiting restrictions, 18% and their families were successfully recruited. Once enrolled, all participants remained in the study until the final visit (100% retention rate). It is worth noting that one patient passed away before the follow-up, but family members continued to participate in the intervention. Figure 2 provides a visual representation of the participants (patients and families) flowchart.

#### Fidelity to Protocol

The intervention phase was executed in strict accordance with the study protocol. A 100% adherence rate was sustained across all screening procedures, ensuring that initial contact was established within 24 hr of each patient’s admission for all four participating families. However, it is worth noting that fidelity to the protocol displayed some variability, spanning from 94% during Phase 1 to 78% during Phase 3. Notably, deviations from the protocol primarily manifested during the briefing and debriefing sessions, with full interprofessional team engagement inconsistently achieved. In terms of protocol adherence during the follow-up phase, fidelity was 54%. This was primarily due to limitations in the CNS’s ability to establish contact, resulting in successful communication with only one family via telephone.

#### Cost Estimation of the Intervention

During Phase 1, the duration of family meetings ranged from 30 to 80 min (*Mdn* = [60], interquartile range [*IQR*] = [40]). In Phase 2, the meetings spanned from 32 to 60 min (*Mdn* = [44], *IQR* = [8]). Phase 3 witnessed family meetings lasting from 5 to 45 min (*Mdn* = [34], *IQR* = [2]).

#### Family Outcomes

**Figure 3.**
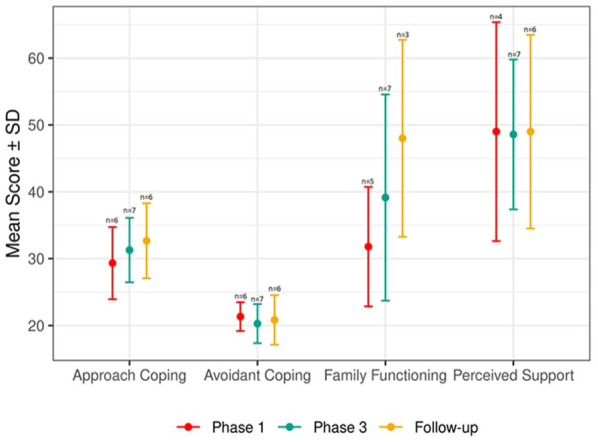
Efficacy Trends for Family Outcomes

The limited efficacy of the SAFIR^©^ intervention on Family Functioning, Family Perceived Support, and Family Coping (evaluated with BRIEF-COPE), during Phase 1, Phase 3, and the follow-up period is presented in Figure 3. Trends indicating improvement subsequent to the intervention are discernible in the domains of Approach Coping and Family Functioning.

### Qualitative Findings

**Figure 4.**
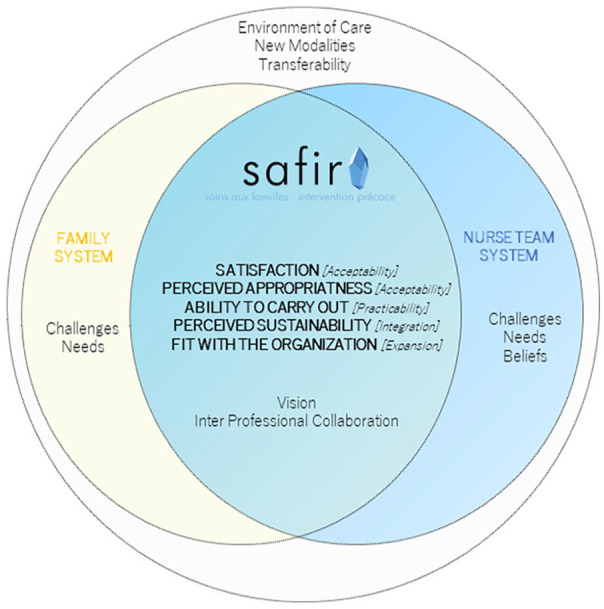
Synthesis of the Qualitative Results.

Six semi-structured interviews were conducted, representing 3 hr and 449 coded segments for analysis. The qualitative content analysis, incorporating both inductive and deductive approaches, revealed five overarching categories: *(a) satisfaction with the intervention, (b) perceived appropriateness, (c) ability of the provider to carry out the intervention, (d) perceived sustainability, and (e) fit with the organization goals and culture* and nine subcategories: *(a) challenges regarding the intervention, (b) family needs, (c) environment of care, (d) beliefs, (e) needs of the HCPs, (f) transferability, (g) new modalities, (h) vision, and (i) interprofessional collaboration*. Figure 4 presents a synthesis of the qualitative results.

### Satisfaction With the Intervention

All family members reported positive experiences with the intervention, emphasizing the exceptional listening skills, attitudes, and availability of the intervention providers. One participant said “It was a welcome ‘break’ from the stress of the situation.” [FAM-003]

The intervention was deemed meaningful, facilitating understanding of the situation, and coping with uncertainty. It provided a platform for family to share experiences, express emotions, and provide support from competent health care professional. One participant noted that the research questionnaire helped putting emotions into perspective and being awareness of own resources. Importantly, receiving future-oriented advice and contact information of other peer-support organizations was highlighted as valuable. The CNS and NMs also echoed the benefits of the intervention, acknowledging the intervention to facilitate partnering with families, preparing for future transitions, and supporting families at each phase of the intervention.

Family members acknowledged the challenge of openly discussing family-related and illness-related issues with unfamiliar HCP (Challenges regarding the intervention). One participant said that the main challenge was “to speak openly to a stranger about all aspects of the patient’s family and illness” [FAM-001]. However, they recognized that the intervention allowed them to address prior challenges and appreciate the need for family support during hospitalization and rehabilitation. Some found it difficult to maintain a positive attitude with their loved ones. Participants also cited difficulties in accepting help and finding time to attend the family consultations and to complete the research questionnaires. The CNS has reported occasional research-related difficulties, such as asking families to fill in questionnaires before a therapeutic relationship has been established. The COVID-19 pandemic emerged as a significant constraint, resulting in ceasing research activities and restricting family visits.

### Perceived Appropriateness

The CNS mentioned difficulties in using intervention tools while simultaneously interacting with family members at the beginning of the study, but these challenges were resolved with experience. The NMs unanimously recognized SAFIR^©^’s value in addressing family needs and noted that it was the only time where families received specific attention. They deemed the intervention relevant not only for the studied population but also for other patients in the hospital.

The intervention provided a platform for families to express all types of emotion, including the most intense (the family needs). NMs emphasized that the intervention was developed to address the specific needs of this population, as hospitalization is a time of crisis characterized by intense emotions and questions.

### Ability of the Provider to Carry Out the Intervention

The CNS highlighted several positive elements regarding the execution of the intervention, including proficiency in utilizing the toolbox, growing confidence in establishing partnerships with families, and the facilitator role in understanding patient situations and family resources:

uh I’m always moved when I talk about it, uh that [. . .] during these interviews I’ve been able to guide . . . families to identify their resources, to give them some tools on how to take care of themselves, [. . .] So it’s huge! [CNS-001]

The briefing sessions with the interprofessional team and the availability of specific rooms for family meetings were viewed positively. The NMs found the pre-intervention training session useful in enabling the CNS to understand the methodology. From an educational and clinical perspective, the NMs found the intervention easy to implement. Some participants stressed the importance of maintaining creativity during interventions, while others noted constraints on conducting daily interventions. NMs believed that the provider was well-suited for the intervention due to expertise in the field and training in FSN.

#### The Environment of Care

The NMs highlighted the challenges associated with the organization of the nursing care and logistics (e.g., place where interview would take place). They also highlighted the difficulty of making members of their nursing team available for research. The CNS identified the challenge of having to provide the intervention outside working hours to comply with the study protocol.

#### The Beliefs

CNS considered time constraints a potential barrier for the health care team, although the intervention was seen as a time-saving measure and an efficiency enhancer. NMs expressed some skepticism about having a dedicated intervention provider regarding the uncertainty surrounding patient situations.

### Perceived Sustainability

CNS emphasized the integration of family care into the role’s specifications and viewed the intervention as a paradigm shift requiring education, mentoring, clinical expertise, and knowledge of the health care system. NMs highlighted the intervention’s value in the care structure and advocated for its integration into patient electronic files. They suggested SAFIR^©^ to become a departmental project with universal applicability, including revisions for flexibility and early-stage integration; “[. . .] but SAFIR gives us something more structured and systematic” [NM-005].

#### The Needs of HCPs

CNS highlighted the need for education in FSN conversation tools, such as genogram and ecomap, while NMs identified health care team needs such as family systems interventions education, continuous information, and interprofessional collaboration.

#### Transferability

CNS expressed certainty in the intervention’s transferability, while NMs showed interest in extending the intervention to other populations.

#### New Modalities

CNS stressed the need for in-person intervention, while NMs, based on pandemic experiences, requested new technologies to support families, regardless of the context.

### Fit With the Organization Goals and Culture

CNS viewed the evolution of the nursing role as an opportunity for implementing family interventions like SAFIR^©^. NMs noted that the project aligned with the department’s goals and reported that the health care team was well-informed about its impact on families and their roles.

#### The Vision

CNS believed that family interventions and interprofessional collaboration were essential elements for nursing discipline development. All participants advocated for family inclusion in patient decisions, highlighting the mission for family support in crisis situations. They emphasized the need for SAFIR^©^ to become an integral part of routine care and clinical managers’ education.

#### The Interprofessional Collaboration

CNS highlighted the benefits of interprofessional collaboration during the intervention, including emotional and informational sharing with families and health care teams, enhanced care coordination, and collaboration with liaison nurses. They noticed that the collaborative work remains a challenge: “Well, there’s still a certain amount of multidisciplinary missing” [NM-001]. Participants perceived family intervention as inherently interprofessional, emphasizing reciprocity and interrelations between systems, with both families and health care teams as partners. This collaboration fostered solidarity, open-mindedness, meaningfulness, and self-confidence.

## Discussion

This study aimed to assess the feasibility of our newly developed intervention SAFIR^©^. To the best of our knowledge, this is the first study to test an early family intervention for patients with ABI. Based on our results, the SAFIR^©^ intervention appears to be feasible in our context and acceptable to families and intervention provider. Recruitment was stopped during the outbreak of the SARS-COV2 pandemic when families’ access to hospital was restricted. When reinitiated, recruitment remained limited. However, when families could be contacted and agreed to participate, they remained in the study until completion, as shown by a high retention rate. Decreased adherence rate in the follow-up phase was mainly due to the difficulty of remote management. Barriers to remote management implementation include the organization and coordination of telephone contacts, accessibility, and cooperation. In view of improvement, the monitoring component should be reviewed to limit these barriers (Otto & Harst, 2019). The high average time spent with families shows the importance of implementing this intervention and confirms families’ need for support. These findings are consistent with the needs expressed by families of acute stroke survivors, particularly in terms of professional support and the need for information (Lou et al., 2015). Time spent with patients appears to be in line with other studies (Mores et al., 2018; Rasmussen et al., 2021). Similar recruitment challenges have been reported in feasibility studies in acute care contexts, such as intensive care (Mitchell et al., 2017) and rehabilitation (Rasmussen et al., 2019), where high retention rates were observed despite poor recruitment rates, attributed to factors like family fatigue and time constraints. However, it has been well-established that families of patients in such situations require substantial support from health care providers (de Goumoëns et al., 2018). Therapeutic relationships, a crucial element, demand time and resources from both health care providers and families (Fisher et al., 2020). To address this gap, the SAFIR^©^ intervention may benefit from revisions facilitating systematic integration into daily routines based on duration and frequency (de Goumoëns et al., 2022). Moreover, family members and the CNS emphasized the importance of providing a dedicated time and place for families to “break” during crisis situations, underscoring the need to consider contextual factors in future intervention implementations. Our results demonstrated strong adherence to the protocol, except for research measures that proved challenging to complete during family meetings, while therapeutic relationships were formed.

The acceptability of the intervention was deemed favorable by family members, the CNS, and NMs. Key factors supporting this result included the CNS’s competencies, congruence between the intervention and family needs, and alignment with the department’s vision. Notably, the professional attitude and skills of the CNS emerged as crucial factors, highlighting the importance of provider leadership skills for successful implementation (Gettrust et al., 2016). However, international recommendations for family interventions advocate for collaboration (IFNA, 2017). We therefore encourage the SAFIR^©^ intervention to involve a wider interprofessional team. Interprofessional collaboration’s value lies in its ability to provide a comprehensive perspective on the patient and family’s needs through profession-specific axes. Through complementarity, this approach enhances support for individuals with ABI and their families (Carron et al., 2021). It should also promote care continuity and information sharing, with a more frequent and continuous presence around the patient and family. The evolution of health care disciplines, including new roles and responsibilities, will support interprofessional “champions” to lead family intervention and improve interprofessional work through competencies (Tracy et al., 2023). The benefits of interprofessional family interventions, such as increased creativity and improved interprofessional communication, have already been demonstrated (Naef et al., 2020). Further improvements in structured and systematic inclusion of all interprofessional team members are needed, as suggested by NMs. Interprofessional collaboration is pivotal when working with families in clinical neurosciences (Ehrlich et al., 2022). In addition, the CNS reported the intervention’s contribution to empowerment and continuous learning, highlighting the mutual benefits of a family systems approach (Meixner et al., 2017).

Families and patients’ relatives reported positive experiences with the SAFIR^©^ intervention, perceiving it as an empathetic moment and an opportunity to take respite during a challenging period of life. It allowed them to gain a better understanding of the events related to the patient’s situation. Prior research has shown that involving families in care through effective and integrated communication, as well as including them in patient-related decisions, enhances the quality of care in the acute phase (Mackie et al., 2019). Concurrent to the findings of the study by Roberts et al., the SAFIR^©^ intervention also appears to provide essential support in addressing the emotions, faith, hope, and medical reality balance, role changes, and adjustment to life disturbance, for which it was designed (Roberts et al., 2021). Interventions focused on families, like SAFIR^©^, offer crucial support during the acute phase, establishing a trust-based relationship between HCPs and families. This relationship, often enduring due to the often-definitive nature of the patient’s injuries, plays a significant role in the family’s life.

Furthermore, the intervention demonstrated an encouraging trend of improved family functioning from baseline to follow-up, as well as an improvement in approaching coping. While perceived support and avoidant coping did not change over time, it is possible that the sample size was insufficient to detect any trend of change.

The sample size of our study was significantly reduced due to pandemic-related visitor restrictions, representing one of the most important limitations. However, because it was a feasibility study, our results remain informative for adaptation of the intervention and planning of further research. Another limitation is related to the homogeneity of the patients’ characteristics restricting our ability to assess the feasibility and acceptability of the SAFIR^©^ intervention for families of individuals with Traumatic Brain Injuries. It would be worthwhile to extend the feasibility testing of SAFIR to these families prior to engaging in a larger trial. One notable strength of our study was its systemic approach to early family intervention, considering both human and environmental factors. This approach enabled the identification of factors that facilitate or hinder the implementation and sustainability of the intervention.

## Conclusion

The findings of this study indicate that the SAFIR^©^ intervention is feasible in a well-prepared clinical setting and has been well-received by families, the intervention provider, and the NMs. Notably, the results highlight a desire for improved interprofessional collaboration, including greater involvement of family members. Consequently, there is a need for a significant revision of the SAFIR^©^ intervention to include a broader interprofessional team. The coordination of the intervention, including recruiting families and coordinating the interventions with care and family’s availability, also need review to reach families requiring support more effectively. In addition, further testing in a large-scale study is warranted before full implementation in clinical practice can be considered. Future research should also explore the role of the CNS to clarify the interpretation of their scope of practice in relation to standard care practices.

## Supplemental Material

sj-doc-1-jfn-10.1177_10748407241270034 – Supplemental material for Caring for Families of Patients With Acquired Brain Injury Early During Hospitalization: A Feasibility Study of the SAFIR© Intervention Supplemental material, sj-doc-1-jfn-10.1177_10748407241270034 for Caring for Families of Patients With Acquired Brain Injury Early During Hospitalization: A Feasibility Study of the SAFIR© Intervention by Véronique de Goumoëns, Krystel Bruyere, Dionys Rutz, Jérôme Pasquier, Yann-Olivier Bettex, Krystel Vasserot, Philippe Ryvlin and Anne-Sylvie Ramelet in Journal of Family Nursing
